# A Bayesian Negative Binomial Hierarchical Model for Identifying Diet–Gut Microbiome Associations

**DOI:** 10.3389/fmicb.2021.711861

**Published:** 2021-10-07

**Authors:** Alma Revers, Xiang Zhang, Aeilko H. Zwinderman

**Affiliations:** ^1^Department of Epidemiology and Data Science, Amsterdam University Medical Center, Amsterdam, Netherlands; ^2^Theoretical Biology and Bioinformatics, Department of Biology, Utrecht University, Utrecht, Netherlands

**Keywords:** associations, Bayesian, diet, hierarchical model, microbiome data

## Abstract

The human gut microbiota composition plays an important role in human health. Long-term diet intervention may shape human gut microbiome. Therefore, many studies focus on discovering links between long-term diets and gut microbiota composition. This study aimed to incorporate the phylogenetic relationships between the operational taxonomic units (OTUs) into the diet-microbe association analysis, using a Bayesian hierarchical negative binomial (NB) model. We regularized the dispersion parameter of the negative binomial distribution by assuming a mean-dispersion association. A simulation study showed that, if over-dispersion is present in the microbiome data, our approach performed better in terms of mean squared error (MSE) of the slope-estimates compared to the standard NB regression model or a Bayesian hierarchical NB model without including the phylogenetic relationships. Data of the Healthy Life in an Urban Setting (HELIUS) study showed that for some phylogenetic families the (posterior) variances of the slope-estimates were decreasing when including the phylogenetic relationships into the analyses. In contrast, when OTUs of the same family were not similarly affected by the food item, some bias was introduced, leading to larger (posterior) variances of the slope-estimates. Overall, the Bayesian hierarchical NB model, with a dependency between the mean and dispersion parameters, proved to be a robust method for analyzing diet-microbe associations.

## 1. Introduction

Human gut microbiota composition plays an important role in human health and has been linked to diabetes, cardiovascular, and mental health diseases (Wu et al., 2011; Morgan et al., 2012; Lynch and Pedersen, 2016; Singh et al., 2017). Targeting gut microbiota composition is, therefore, considered a promising treatment for several diseases (Smits et al., 2013). For example, fecal microbiota transplantation (FMT) has been used to treat patients with insulin resistance and metabolic syndrome (Vrieze et al., 2012). In contrast to FMT, long-term diet intervention is a much less invasive intervention that can shape human gut microbiota and influence human health (Wu et al., 2016; Singh et al., 2017). Therefore, many studies focus on discovering links between long-term diets and gut microbiota composition, and examples of recently published studies are Wu et al. (2019), Han and Xiao (2020), Tomova et al. (2020), and Molino et al. (2021).

One way to study the relationship between diet and gut microbiota is through evaluating diet-microbe associations. This type of study is often based on a cohort or population in which both dietary intake and gut microbiome are measured extensively. For example, a substudy of the Health Life in an Urban Setting (HELIUS) study collected both dietary intake and gut microbiome data in over 1,000 subjects (Stronks et al., 2013). In a previous study, the effect of ethnicity and dietary habits on the gut microbiome was investigated (Deschasaux et al., 2018).

In a previous study, we showed that it is challenging to perform statistical evaluation of diet-microbe associations (Zhang et al., 2019). Microbiome data are often generated by targeted sequencing of the 16S ribosomal RNA (rRNA) gene. The sequencing reads are clustered into operational taxonomic units (OTUs) based on the sequence similarities. A simplified interpretation is that an OTU represents a microbe. The microbiome data are often represented as a frequency matrix, in which every entry is the number of times an OTU is observed in a sample. In general, microbiome data have the following features: (i) Library sizes, the total count in a sample, can vary by orders of magnitude across samples (McMurdie and Holmes, 2014). (ii) Counts of a single OTU are often over-dispersed. Microbiome data often have excess zero counts, meaning that a single OTU is only present in a small number of fecal samples (Kaul et al., 2017). These zero counts can be due to either biological absence of a microbe or insufficient sequencing. (iii) There is a hierarchical structure between the OTUs (represented by phylogenetic relationships).

The phylogenetic relationships between OTUs are based on OTU sequence similarities. Every OTU can be assigned to a series of taxonomic identities at levels, such as kingdom, phylum, class, order, family, and genus. These relationships reflect the evolutionary relationships between the OTUs. Closely related OTUs usually have a similar biological function and are likely to be affected similarly by food consumption (Washburne et al., 2018). Hence, the phylogenetic relationships provide prior knowledge about how the OTUs are related.

Not every level within the taxonomic identities is as informative. For the higher levels, kingdom, phylum, and class, the assumption about similar biological functions is not likely valid. At the lower levels, the number of OTUs per genus, for example, is small. Although they might share a similar biological function and are likely to be affected similarly by food consumption, having a low number of OTUs can lead to convergence issues. Furthermore, not every OTU has the entire taxonomy identified. For example, only 51% of the OTU has a fully identified hierarchy in the HELIUS data.

When performing per-feature analysis, a high number of false positives can be expected. Adjustments to control the false discovery rate (FDR) are then needed. A way to control the FDR is by using shrinkage of the estimated effects toward zero. However, there is always a trade-off, as this shrinkage toward zero will increase the false-negative rate. Therefore, we propose to use a slightly more informative shrinkage toward a phylogenetic family average.

In this study, we aimed to incorporate the phylogenetic relationships between the OTUs into diet-microbe association analysis. A direct way to incorporate the relationships between OTUs is by using hierarchical models. We constructed our hierarchical model with a fully Bayesian approach. As a result, we developed a Bayesian hierarchical negative binomial model (BHM) for microbiome data analysis. Following others, we use a regularization of the dispersion parameter of the negative binomial (NB) distribution by assuming a linear mean-dispersion association (Love et al., 2014).

## 2. Materials and Methods

Microbiome count data consist of observations of 1…*i*…*N*_*subj*_ subjects on 1…*j*…*N*_*otu*_ OTUs. The frequency matrix of the OTU counts **Y**, each row represents a subject, and every column represents an OTU, where *Y*_*ij*_ represents how many times OTU *j* is observed in the subject *i*. The total counts (or sequencing depth) in subject *i* is represented by $z_{i}=\overset{N_{otu}}{\underset{j}{\sum }}Y_{ij}$. The phylogenetic family of all OTUs is assumed to be known.

The food frequency questionnaire (FFQ) data are represented as a matrix **X** with *N*_*subj*_ rows and *N*_*diet*_ columns, where *X*_*ik*_ represents the frequency of dietary variable *k* in subject *i*. For notation simplification, as we only use univariate analyses, a single column of **X** is referred to as **x**, where **x** is a vector with length *N*_*subj*_. The data structure are illustrated in Table 1.

**Table 1:** The data structure of the microbiome data.

	***OTU*_1_**	**⋯**	***OTU*_*j*_**	**⋯**	***OTU*_*N*_*otu*__**	**Total counts**	**Food item score**
Phylogenetic family	1	⋯	*f*	⋯	*N*_*family*_		
*Subject*_1_	*y*_11_	⋯	*Y*_1*j*_	⋯	*Y*_1*N*_*otu*__	*z*_1_	*x*_1_
⋮	⋮		⋮		⋮	⋮	⋮
*Subject*_*i*_	*Y*_*i*1_	⋯	*Y*_*ij*_	⋯	*Y*_*i* *N*_*otu*__	*z*_*i*_	*x*_*i*_
⋮	⋮		⋮		⋮	⋮	⋮
*Subject*_*N*_*subj*__	*y*_*N*_*subj*1__	⋯	*y*_*N*_*subjj*__	⋯	*y*_*N*_*subj*_ *N*_*otu*__	*z*_*N*_*subj*__	*x*_*N*_*subj*__

Our model consists of three levels. In the first level, the OTU counts are modeled. We choose to model the counts *Y*_*ij*_ (OTU *j* subject *i*) using a NB distribution. The NB distribution has two parameters, the mean μ and a dispersion parameter ϕ:


$$\begin{array}{l} Y_{ij}\sim \text{NB}\left(\mu_{ij},\phi_{j}\right) \end{array}$$

Using a logarithmic link function, the mean μ_*ij*_ is specified as:


$$\begin{array}{l} \text{log}\left(\mu_{ij}\right)=\alpha_{j}+x_{i}\beta_{j}+\text{log}\left(z_{i}\right) \end{array}$$

Here, α_*j*_ is the intercept of the model for OTU *j*, *x*_*i*_ is the standardized FFQ score of a single food item of subject *i*, and β_*j*_ is the slope of the regression model for OTU *j*. The offset is the log of the total count of subject *i* (*z*_*i*_), to account for the uneven sequencing depth between subjects.

In the second level, we take the phylogenetic family into account and shrink the posterior estimate parameters of the slope and intercept toward the phylogenetic family average. Let *OTU*_*j*_ be a microbe of the phylogenetic family *f*. We then assume that the intercepts and slopes of OTUs in family *f* follow a bivariate normal distribution with mean μ_α_*f*__, μ_β_*f*__, and covariance matrix Σ_*f*_.


$$\begin{array}{l} \left(\begin{array}{l} \alpha_{j}\\ \beta_{j} \end{array}\right)\sim N(\mu_{f},\Sigma_{f}),\text{with}\\ \;\mu_{f}=\left(\begin{array}{l} \mu_{\alpha_{f}}\\ \mu_{\beta_{f}} \end{array}\right)\text{and}\\ \;\Sigma_{f}=\left(\begin{array}{cc} \sigma_{\alpha_{f}}^{2} & \omega \sigma_{\alpha_{f}}\sigma_{\beta_{f}}\\ \omega \sigma_{\alpha_{f}}\sigma_{\beta_{f}} & \sigma_{\beta f}^{2} \end{array}\right). \end{array}$$

The covariance matrix Σ_*f*_ consists of family-specific variances ($\sigma_{\alpha_{f}}^{2},\sigma_{\beta_{f}}^{2}$) and correlation parameter ω. This correlation parameter describes the relationship between slope β and intercept α. The correlation parameter is independent of family *f*, as some families may only have a small number of members.

In the third level, we specify prior distributions. For each μ__α_*f*_ and μ__β_*f*_, we use a normal distribution as prior distribution with:


$$\begin{array}{l} \begin{array}{c} \mu_{\alpha_{f}}\sim N\left(\mu_{\alpha },\sigma_{\alpha }^{2}\right)\\ \mu_{\beta_{f}}\sim N\left(\mu_{\beta },\sigma_{\beta }^{2}\right) \end{array} \end{array}$$

For the hyperparameters μ_α_, μ_β_, $\sigma_{\alpha }^{2}$, and $\sigma_{\beta }^{2}$, we specify as much as possible non-informative priors:


$$\begin{array}{l} \begin{array}{c} \left(\mu_{\alpha },\mu_{\beta }\right)\sim N\left(0,1\right)\\ \left(\sigma_{\alpha },\sigma_{\beta }\right)\sim \text{exponential}\left(1\right) \end{array} \end{array}$$

In addition, we assume a mean-dispersion relationship between the average of the NB distribution mean of OTU *j* over all subjects and the dispersion ϕ_*j*_ of OTU *j*. Following the specification used by Love et al. (2014), the dispersion ϕ_*j*_ is drawn from a lognormal distribution with mean μ_ϕ_*j*__ and variance $\sigma_{\phi }^{2}$.


$$\begin{array}{l} \phi_{j}\sim \text{lognormal}\left(\mu_{\phi_{j}},\sigma_{\phi }^{2}\right) \end{array}$$

where μ_ϕ_*j*__ is specified as $\mu_{\phi_{j}}=\frac{a_{1}}{\left(\overset{N_{subj}}{\underset{i}{\sum }}\mu_{ij}\right)/N_{subj}}+a_{0}$.

The expected range of values for the parameters *a*_1_ and *a*_0_ is unknown. Large ϕ values and values close to zero are what we try to prevent by using this regularization. Therefore, for the parameters *a*_1_, *a*_0_ we assume the following priors (and for $\sigma_{\phi }^{2}$ a non-informative prior):


$$\begin{array}{l} \begin{array}{c} \left(a_{1}a_{0}\right)\sim N\left(0,1\right)\\ \;\sigma_{\phi }\sim \text{exponential}\left(1\right) \end{array} \end{array}$$

We implemented our model in the Stan probabilistic programming language, which estimates the posterior distributions for the parameters of interest by using Hamiltonian Markov Chain Monte Carlo (HMC) (Carpenter et al., 2017). Stan was used with the default settings: 4 chains with 1,000 warm-up iterations and 1,000 samples of the posterior distributions per chain to calculate summarizing statistics. No alterations on the default values of the maximum allowed tree-depth or adapt delta parameter were needed for our analyses as they all reached convergence with these settings. A thinning of two was used for memory reasons.

### 2.1. Simulation Study Design

A simulation study was conducted to assess the performance of the proposed method and to compare the performance with other methods. Our simulation study consisted of a total of 18 scenarios. The 18 scenarios included different numbers of OTUs and subjects. We simulated 10, 100, or 200 OTUs from 2 families and *N*_*subj*_ = 50, 250, or 500 subjects. In total, there are nine combinations of OTUs and subjects, giving a total of nine scenarios. On top of the nine scenarios, we added either over-dispersion or under-dispersion to the simulated count data. As zeros are common within microbiome data, we simulated datasets including zeros as well. In the end, our simulation study contained 18 scenarios. In each scenario, we generated 100 datasets, resulting in a total of 1,800 datasets.

We generated data in all scenarios according to the following procedure (simulation framework can be found in the Supplementary Material):

(i) For all OTUs, one of two families was randomly selected. The intercept and slope of family 1 were drawn from a normal distribution with means μ_α_ = 2, μ_β_ = 0, and SD σ_α_ = σ_β_ = 0.1. For the intercept and slope of family 2, a normal distribution with means μ_α_ = 2, μ_β_ = +1 or −1 and SD σ_α_ = σ_β_ = 0.1.
(ii) The simulated food item scores **x** for all subjects were drawn from a normal distribution, with a mean of 0, +1, or −1 (μ_*x*_), randomly chosen. The food items scores (*x*) can be skewed distributed, and, therefore, in each scenario in 50 datasets, food item scores for all subjects were squared to simulate a skewed distribution.
(iii) The OTU-specific intercepts and slopes were drawn from a bivariate normal with the means of the appropriate families, a SD of 0.1 (both intercept and slope) and a negative Pearson correlation of −0.7.
(iv) The OTU count data (**y**) were simulated based on **x** and the simulated intercept and slope. We simulated count data with over-dispersion and with under-dispersion. For the counts with over-dispersion, we used a NB distribution to generate counts, with a log mean equal to the intercept plus slope times x. A dispersion was drawn from a lognormal distribution with a mean of 0.1/intercept and a SD of 0.1.

To create under-dispersed data, we used a Poisson distribution, with log mean equal to the intercept plus slope times x. To create under-dispersion and inflation of zero counts, the top 20% of the counts were set to the average OTU count or to zero (A summary of the data characteristics of the simulated datasets can be found in Supplementary Material).

We compare results of our BHM with results of an existing implementation of the NB model (GLM.NB) in the MASS packages of R (Venables and Ripley, 2002) and with a BHM without using the phylogenetic hierarchy. For the BHM without using the phylogenetic hierarchy, we assumed that all slopes and intercepts were drawn from a single bivariate normal distribution (for the full specification, see Supplementary Material).

As for performance measurements of the methods, we calculated (i) the mean squared error (MSE) between the true slopes and the estimated slopes by the various methods, (ii) the bias between the true slopes and the estimated slopes, (iii) the coverage of the 95% CIs defined as the number of times the true slope was within the 95% CI of the estimated slope, and (iv) the false discovery rate (FDR), defined as the number of times the true slope was zero divided by the number of false positives as given by a p-value below 0.05.

### 2.2. Healthy Life in an Urban Setting Data

Of the HELIUS data, we only included subjects with a full set of food item scores and OTUs with positive abundance in at least 20% of the subjects from the HELIUS study. We also only selected OTUs that are part of an identified phylogenetic family with at least four other OTUs. The set of OTUs was split into six batches, ordered by the average abundance of the phylogenetic families. There were a total of 52 food items; food items scores were standardized. A more detailed description of the data is available in Dekker et al. (2011).

We compared our BHM with NB regression implemented in the R package edgeR (Robinson et al., 2010), with GLM.NB and with a BHM without using the phylogenetic hierarchy for the HELIUS data. With edgeR, OTU counts were modeled by NB distribution as well. We first estimated an OTU-specific dispersion, by running the estimate Disp function, as implemented in the edgeR package in R. Next, the associations between the food items and the OTU counts were quantified by the glmFit function, of the edgeR package too. The offset was for all methods defined as the log(total counts).

## 3. Results

In this section, we first present the simulation study results, in which we compared our Bayesian model with other methods. In the second subsection, we present the results of the HELIUS study diet-microbiome data.

### 3.1. Simulation Results

**Figure 1:**
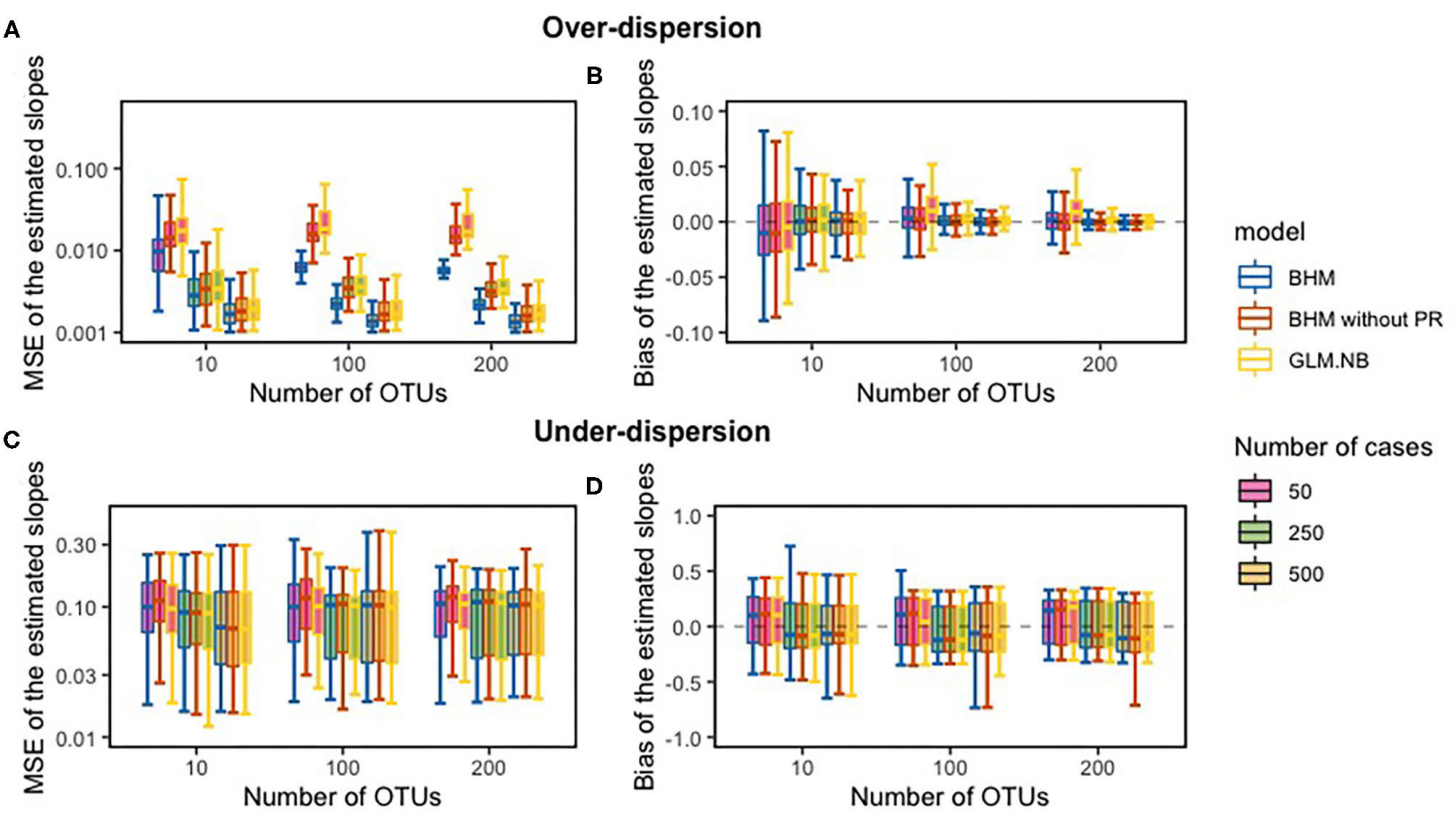
The comparison between the true slope and the estimated slope of the simulation study; the mean squared error (MSE) **(A,C)** and bias between the slopes **(B,D)**. PR, phylogenetic relationships.

Figure 1 displays MSE and bias of the estimated slopes for both our BHM, with and without the phylogenetic structure, and of the NB regression model. The FDR results and the bias and MSE for the zero-inflated standard NB regression model and Poisson model can be found in Supplementary Material. As expected, if over-dispersion was present in the data, for both the BHMs and the standard NB regression model (referred to as GLM.NB) bias of the slope-estimates was close to zero and decreased with increasing sample size and with an increasing number of OTUs. MSE of the slope-estimates decreased with increasing sample size; MSE of the BHM with phylogenetic structure was smaller compared to the GLM.NB and the BHM without the phylogenetic structure when over-dispersion was present in the count data. This smaller MSE for the BHM method was not found when under-dispersion was present in the count data; then, the MSE of the slope-estimates was comparable between methods. Coverage of the 95% CI with the count data with over-dispersion was around 95% when the sample size was 100 or larger, but it was slightly higher with samples of only 10 subjects. With count data with under-dispersion, coverage of the 95% CI was lower for the BHM method.

**Figure 2:**
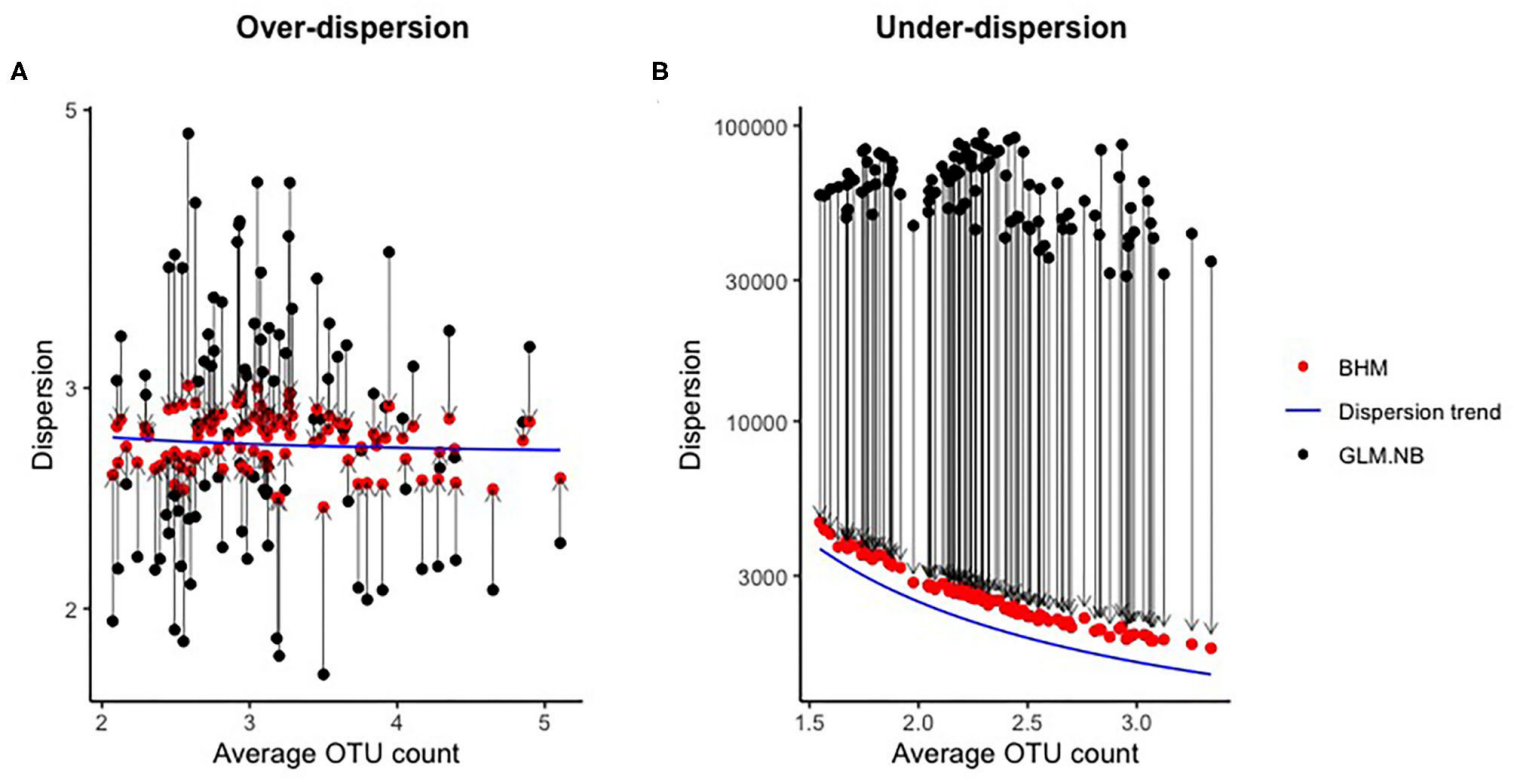
Effect of regularization of the dispersion parameter in the simulation study. Every black-red bot represents a combination of the estimated dispersion parameter of the GLM.NB method (black) and the posterior mean of the BHM (red) method for a single OTU. The blue line is the dispersion trend as estimated by the BHM method.

In Figure 2, the effect of the mean-dispersion regularization is shown for datasets with over-dispersion and under-dispersion, both for a scenario with 100 OTUs and 250 subjects. By imposing the mean-dispersion relationship, the estimates of ϕ_*j*_ of the standard NB model were clearly shrunken or inflated in the BHM to the mean depending on average OTU count. If under-dispersion was present, ϕ_*j*_ tended to become very large without this regularization. With a very large ϕ_*j*_, the NB distribution approximates the Poisson distribution.

### 3.2. Healthy Life in an Urban Setting Diet—Gut Microbiome Data

After the selection criteria, the HELIUS dataset included 1,201 OTUs, of 25 phylogenetic families, 1,036 subjects, and 52 food items. There were no missing data. The set of OTUs was split into six batches, ordered by the average abundance of the phylogenetic families. Of the 25 families, only the OTUs of families *f__Lachnospiraceae* and *f__Ruminococcaceae* were split into multiple batches. The OTUs of all other included families were in the same batch.

**Figure 3:**
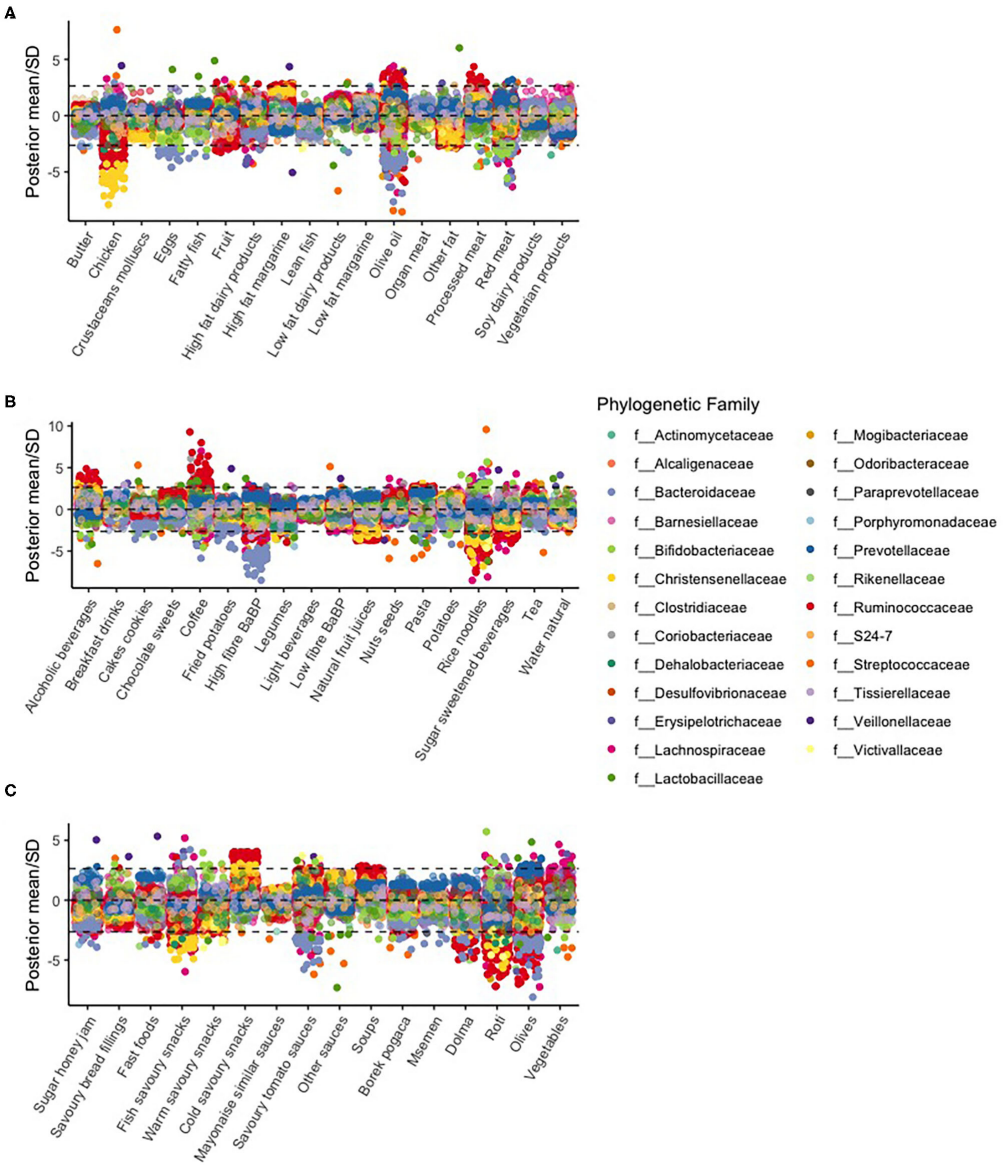
Manhattan plot of various food items [**(A)** butter to vegetarian products, **(B)** alcoholic beverages to water natural, and **(C)** sugar honey jam to vegetables] and associations per OTU. The dotted lines represent a statistical significance threshold (z-score). Because we had six batches, we used the Bonferroni method as a correction factor for multiple testing. Within the batches, we did not correct for multiple testing because the Bayesian hierarchical model already provides shrunken estimates.

For most food items, the estimated slopes were close to zero, and therefore, OTU counts showed no association with food item scores. Figure 3 shows the estimated standardized slopes of OTU counts with food item scores (divided by the estimated SD).

In particular, we found large slopes of OTUs in the *f__Christensenellaceae* family. This family was negatively associated with the chicken, rice noodles, natural fruit juices, and savory fish snacks food item scores. OTUs in the *f__Bacteroidaceae* family were negatively associated with the eggs, olive oil, high fiber bread and breakfast products, savory tomato sauces, and olive food item scores. Coffee was mostly positively associated with OTU counts, most prominently with the *f__Ruminococcaceae* family.

**Figure 4:**
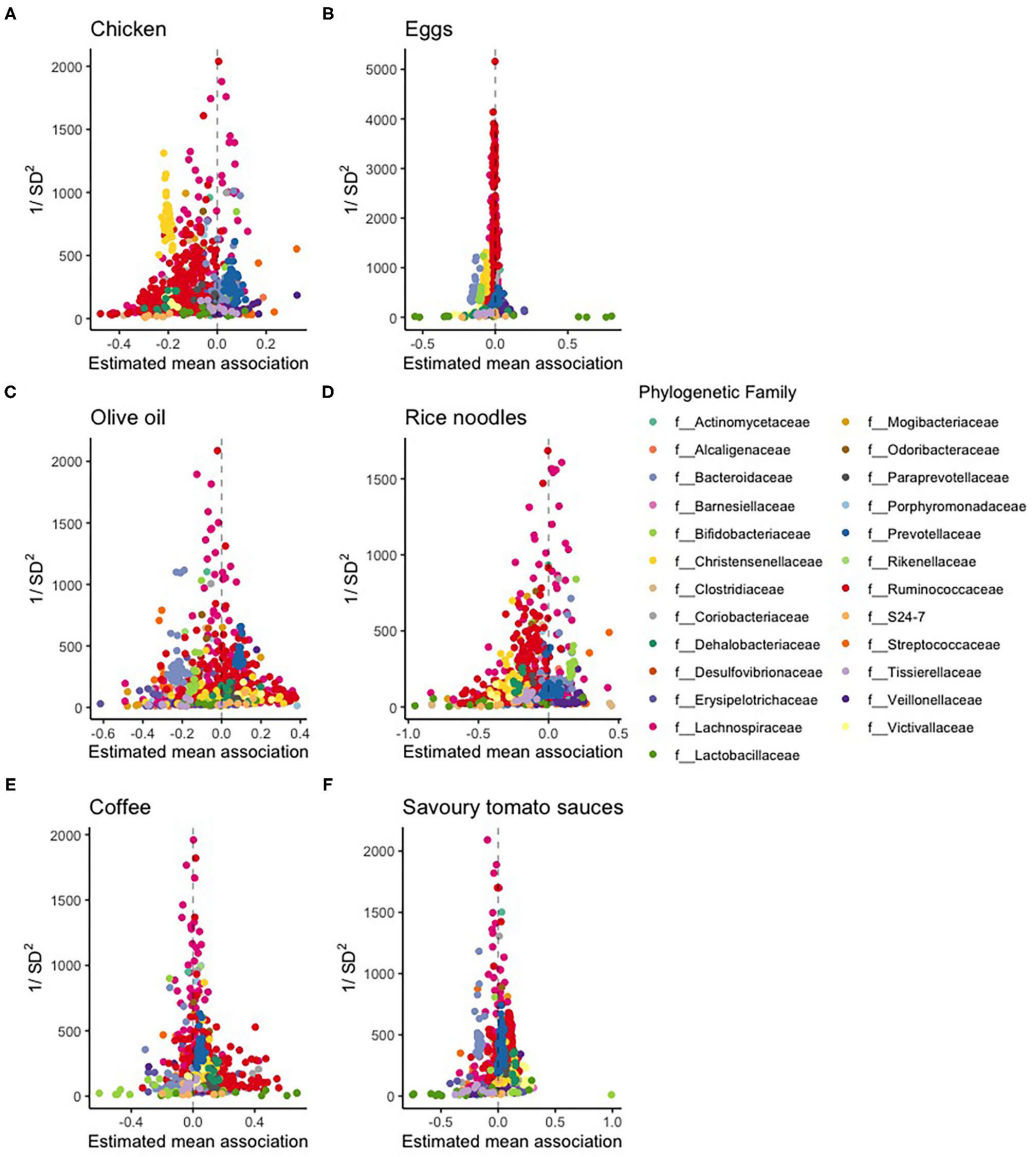
Volcano plots of the diet-microbe associations between the FFQ scores of **(A)** chicken, **(B)** eggs, **(C)** olive oil, **(D)** rice noodles, **(E)** coffee, and **(F)** savory tomato sauces.

The estimated slopes of chicken food scores with the abundances of the OTUs varied between −0.5 and 0.35 (Figure 4). With a unit SD increase of chicken food score, the average *f__Christensenellaceae* abundance decreased by about 20%. Apart from the *f__Christensenellaceae*, single OTUs were also “significantly” associated with an increase in chicken food scores, most clearly the *g_Streptococcus_unclassified* OTU.

The distribution of the estimated of diet-microbe associations of egg food scores showed some outliners of the *f__Lactobacillaceae*; they had, however, large credibility intervals. Coffee consumption had a large positive association with *f__Coriobacteriaceae*.

**Figure 5:**
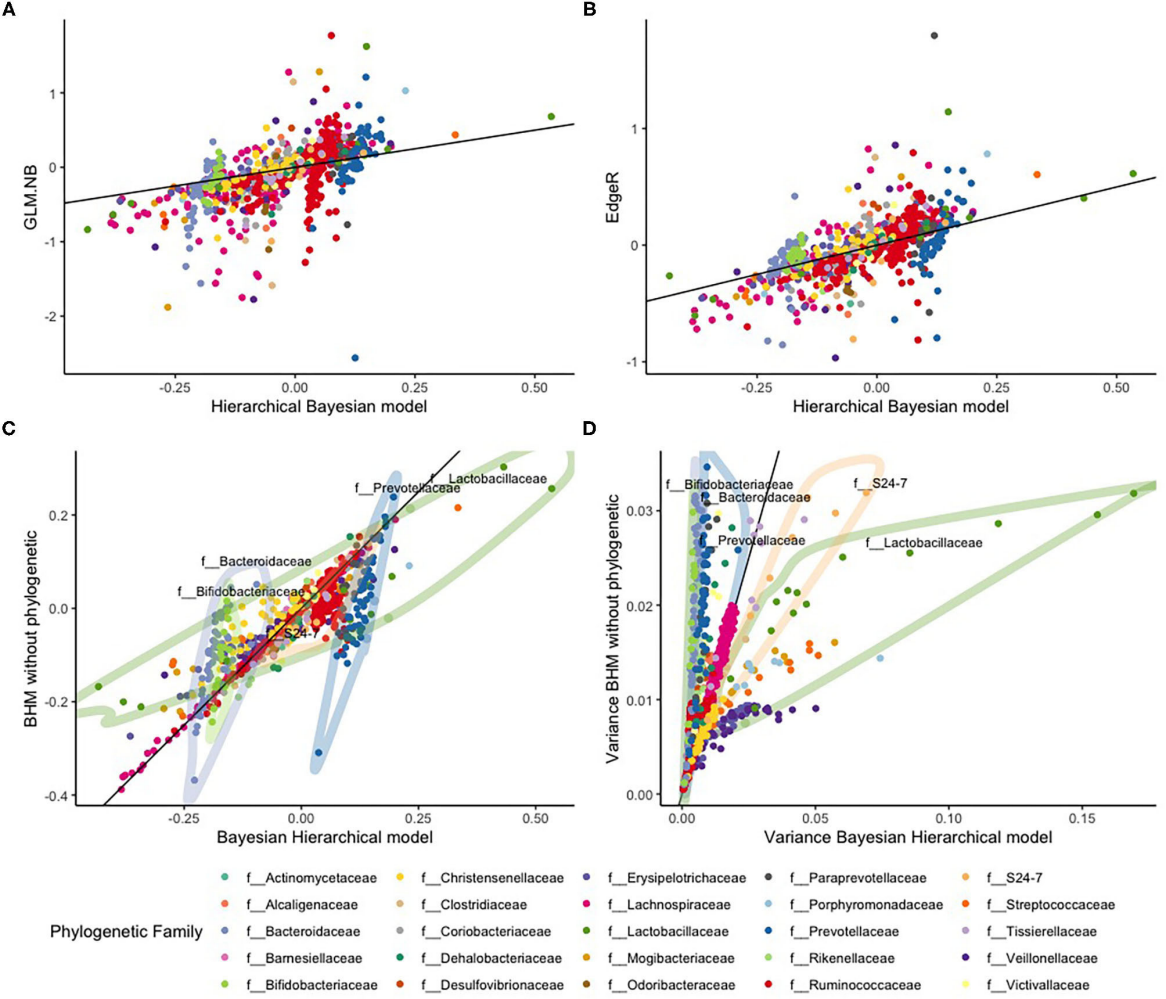
Comparison between the standard NB regression model (GLM.NB), EdgeR, and the Bayesian hierarchical model (BHM) with and without phylogenetic based shrinkage for the HELIUS study data. The top row are the slope estimates from the **(A)** GLM.NB and **(B)** EdgeR methods compared to estimates of our BHM. The bottom row gives a comparison between the BHM with and without the phylogenetic based shrinkage: **(C)** the posterior slope estimates, and **(D)** the variances of these posterior slopes. The black line in all four figures is the diagonal x = y.

We looked at the associations of OTU counts with red meat food scores in more detail and compared parameter estimates of our BHM with those of the standard NB regression model and estimates of EdgeR. Estimated slopes of the standard NB regression model showed much variation with some extreme values, ranging between −2.5 and 2. There were also 12 diet-microbe associations where no unique solution was found with this method. EdgeR slope estimates using the dispersion constraint were smaller on average with lesser variation but still with some extremely large values, ranging between −1 and 1.7. Estimates of our BHM showed lesser variation with much fewer extreme values and slope estimates between −0.3 and 0.8 (see Figure 5).

We also compared slope estimates our BHM model with estimates of a Bayesian NB regression analysis without using the phylogenetic hierarchy. With this model, all parameters are shrunken to a single mean. These results are illustrated in Figure 5. Estimated slopes of most OTUs were comparable, but estimates of OTUs in the *f__Bacteroidaceae* (light purple), *f__Bifidobacteriaceae* (light green), and *f__Prevotellaceae* (dark blue) families showed much less variance according to our BHM model than according to the model without phylogenetic hierarchy. In contrast, estimates of OTUs in the *f__Lachnospiraceae* (green) and *f__S24-7* (orange) families showed larger variance according to our BHM model. Especially with the *f__Lachnospiraceae* (green) family, it can be clearly seen that slopes for OTUs in this family are heteroscedastic with some OTUs having large positive values and some OTUs having large negative values that might indicate that the assumption is not valid that red meat consumption affects the abundances of OTUs in this family in the same way.

## 4. Discussions and Conclusions

The Bayesian hierarchical NB model, with a dependency between the mean and dispersion parameters, proved to be a robust method for analyzing diet-microbe associations. With our model, we analyzed sparse and over-dispersed microbiome data. With the inclusion of the phylogenetic relationships, robust results were possible even with only a three-level structure.

An important limitation of our method is that the method is currently limited by the number of OTUs. We could analyze about 200 OTUs simultaneously on a standard MacBook personal computer. This limitation is the reason that the HELIUS dataset was split into six batches and the use of a higher threshold for selection of OTUs in the HELIUS data (>20% abundance) instead of the more common threshold of 10% abundance. By using six batches, two families of OTUs were split into multiple batches. This limits the advantage of the hierarchical structure. The diet-microbe associations found in our case study are not validated in this study.

Several authors have used the NB distribution for modeling OTU counts (Zhang et al., 2016, 2017; Lee and Sison-Mangus, 2018). Inclusion of the dependency between the mean and dispersion parameter of the NB model has, to our knowledge, not been considered for gut microbiome data. However, it is common for the genomics field (Love et al., 2014). The estimation of the dispersion parameter with a small number of non-zero counts, as with the gut microbiome data, tends to be unstable. The estimate of the dispersion parameter often becomes either very large or close to zero. A solution to this unstable dispersion estimate is to share information across OTUs, by assuming a dependency between the mean and dispersion parameter. This dependency with the mean results mainly in information sharing between OTUs with a similar average abundance, and, thus we assume that OTUs with a similar average abundance will also have a similar variance and, therefore, dispersion. The simulation study showed this mean-dispersion regularization does lead to a slightly worse fit, in terms of coverage of the 95% CI, when under-dispersion is present in the data, compared to a method without this regularization. However, with the microbiome data over-dispersion is very often observed.

The proposed model relies on the assumptions that OTUs of the same phylogenetic family are affected similarly by a food item. This assumption leads to a decrease in the estimated variances of the estimated slopes. Assuming phylogenetic relationships between OTUs are informative is not new. Other methods have been developed using this assumption as well (Zhao et al., 2015; Koh et al., 2017; Xiao et al., 2018). Those methods are, however, not suitable for the problem at hand as they treat the microbiome as an independent variable. In our case, we hypothesized that food intake influences the gut microbiota composition.

In contrast, when a phylogenetic classification is not correct, bias in the estimated slopes may be introduced. This is supported by the increase in some posterior slopes variance when using the phylogenetic-based shrinkage, compared to a BHM without phylogenetic-based shrinkage with the HELIUS data. We, however, only took one method of estimating the phylogenetic tree into account for the HELIUS data. Other tree construction methods might yield different results.

With our model, the phylogenetic relationships are not fully incorporated, because they are only used to group OTUs per family. We did not include genus, order, class, phylum, kingdom, and domain in any way, neither the uncertainty about the phylogenetic relationships themselves. We choose to incorporate the family level, as a lower genus level only has a small number of members per genus, letting OTUs, therefore, share information with a small number of other OTUs. A solution would be to adjust the BHM by adding an extra level. However, in the higher levels, members are more likely to have more variation, making the assumption of similar effect by food consumption more unlikely. We used the family classification, as with the HELIUS data many OTUs were not fully classified. However, using the genus classification can be possible as well with this proposed model. An example of diet-microbe association classification with the genus layer can be found in Supplementary Material.

An alternative to using phylogenetic classification was proposed by Bichat et al. (2020). They used a correlation tree, instead of a phylogenetic tree. This could be translated into classification by using a cut off value. However, the correlations between OTU counts are strongly impacted by the number of subjects with a non-zero OTU count. This makes it likely that most correlations between OTUs are small and noisy. A comparison of the correlation-based grouping and the phylogenetic-based grouping can be found in Supplementary Material.

We currently only used univariate analyses to quantify the association between OTU counts and food item scores, because, at present, we were interested in specific food items and not in food patterns. We did also not adjust for ethnicity or other variables affecting the gut-microbiome composition. It is straightforward, however, to extend our model to a multiple regression model.

## Data Availability Statement

Publicly available datasets were analyzed in this study. This data can be found here: The 16S rRNA gene sequences have been deposited at the European Genome-phenome Archive under study number EGAD00001004106. The FFQs data of this study are available from the study coordinator upon reasonable request.

## Ethics Statement

The studies involving human participants were reviewed and approved by the Academic Medical Center (AMC) Medical Ethics Committee. The patients/participants provided their written informed consent to participate in this study.

## Author Contributions

AR, XZ, and AZ developed the statistical model. AR and AZ developed simulation framework. AR conducted the simulation study and data analysis, and also prepared the first draft. XZ and AZ edited the manuscript. AZ provided the HELIUS study data. All authors contributed to the article and approved the submitted version.

## Conflict of Interest

The authors declare that the research was conducted in the absence of any commercial or financial relationships that could be construed as a potential conflict of interest.

## Publisher's Note

All claims expressed in this article are solely those of the authors and do not necessarily represent those of their affiliated organizations, or those of the publisher, the editors and the reviewers. Any product that may be evaluated in this article, or claim that may be made by its manufacturer, is not guaranteed or endorsed by the publisher.
