# Supplementary material for: A Bayesian Negative Binomial Hierarchical Model for Identifying Diet–Gut Microbiome Associations
Source: Front Microbiol. 2021 Oct 7;12:711861. doi: 10.3389/fmicb.2021.711861 (PMC8529249; doi:10.3389/fmicb.2021.711861)
Supplement: Supplementary file 1 [file Data_Sheet_1.PDF]

Supplementary Material

SIMULATION FRAMEWORK

$$x_{i*} \sim N(\mu_x, 0.1)$$

For every subject a simulated food item score is drawn from a normal distribution with a mean μ_x and a standard deviation of 0.1. The μ_x is 0, 1 or -1 randomly. For datatsets 1 till 50 $x_i = x_{i*}$. For datasets 51 till 100 per scenario the simulated food item score is squared ($x_i = x_{i*}^2$).

$$\mu_{\alpha_f} \sim N(2, 0.1)$$
$$\mu_{\beta_f} \sim N(\mu_\beta, 0.1)$$
$$\begin{pmatrix} \alpha_j \\ \beta_j \end{pmatrix} \sim N(\begin{pmatrix} \mu_{\alpha_f} \\ \mu_{\beta_f} \end{pmatrix} \begin{pmatrix} \sigma_\alpha^2 & \omega\sigma_\alpha\sigma_\beta \\ \omega\sigma_\alpha\sigma_\beta & \sigma_\beta^2 \end{pmatrix})$$

The OTU specific intercept and slope are simulated by first drawing a family specific average from a normal distribution. The family specific slope is randomly drawn with a μ_β of 0, -1 or 1. Next, OTU specific intercept and slope is drawn from a bivariate normal, with standard deviations of 0.1 ($\sigma_\alpha = \sigma_\beta = 0.1$) and a correlation of -0.7.

$$\phi_j \sim \text{lognormal}(0.1/\alpha_j, 0.1)$$
$$y_{ij} \sim \text{NB}(\text{exp}(\alpha_j + \beta_j * x_i), \phi_j)$$

For the scenarios with over-dispersion the counts are drawn from a negative binomial distribution.

$$y_{ij} \sim \text{Poisson}(\text{exp}(\alpha_j + \beta_j * x_i))$$

For the scenarios with under-dispersion the counts are drawn from a Poisson distribution, the top 20% counts are set to the average count to simulated under-dispersion.

FULL SPECIFICATION OF THE BHM WITHOUT PHYLOGENETIC RELATIONSHIPS

$$
\begin{aligned}
y_{ij} &\sim \text{NB}(\mu_{ij}, \phi_j) \\
\log(\mu_{ij}) &= \alpha_j + x_i\beta_j + \log(z_i) \\
\phi_j &\sim \text{lognormal}(\mu_{\phi_j}, \sigma^2_\phi) \\
\mu_{\phi_j} &= \frac{a_1}{(\sum_i^{N_{subj}} \mu_{ij})/N_{subj}} + a_0 \\
\begin{pmatrix}\alpha_j \\ \beta_j\end{pmatrix} &\sim N(\begin{pmatrix}\mu_\alpha \\ \mu_\beta\end{pmatrix} \begin{pmatrix} \sigma^2_\alpha & \omega\sigma_\alpha\sigma_\beta \\ \omega\sigma_\alpha\sigma_\beta & \sigma^2_\beta \end{pmatrix}) \\
(\mu_\alpha\mu_\beta) &\sim N(0,1) \\
(\sigma_\alpha\sigma_\beta) &\sim \text{exponential}(1) \\
\omega &\sim \text{Cholesky LKJ Correlation}(2) \\
(a_1 a_0) &\sim N(0,1) \\
\sigma_\phi &\sim \text{exponential}(1)
\end{aligned}
$$

THE DATA CHARACTERISTICS OF THE SIMULATED STUDY

Table S1. The data characteristics of the simulated study

Number of OTUs	Number of subjects	Mean % zero	Max % zero	Mean difference	Max difference
10	50	14.5	40.0	18.9	704.7
	250	14.9	35.2	444.4	172301.6
	500	14.4	31.6	16262.1	23587293
100	50	14.4	48.0	25.7	5784.4
	250	14.6	36.0	598.7	2372504
	500	14.3	39.2	1165.6	914386.2
200	50	14.5	48.0	35.2	16431.1
	250	14.5	38.8	209.3	572838.6
	500	14.5	36.0	383.7	357129.6

FALSE DISCOVERY RATE PERFORMANCE

A

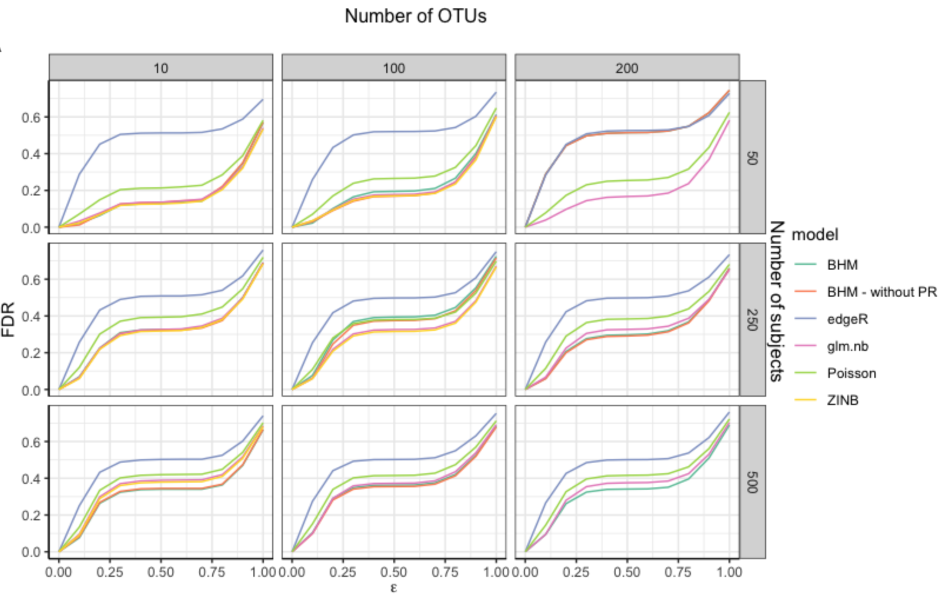

B

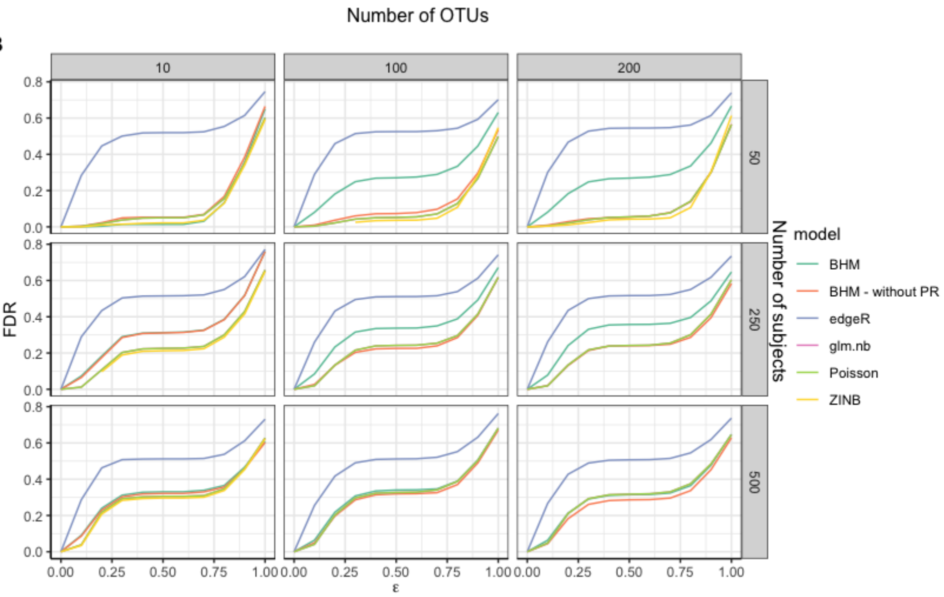

Figure S1. In our data generating mechanism for the simulation study association parameters (β_j) were drawn from a normal distribution. This means most of the associations were close to zero but not exactly equal to zero. To quantify the false discover rate (FDR), we defined all beta's as truly not associated within the interval [-ϵ, +ϵ]. FDR was subsequently calculated as the number of true betas within the interval among the number of estimated betas with p-values less than 0.05. From the graph, we can clearly observe that, when over-dispersion is present **(A)**, the FDR of EdgeR and the Poisson model were much higher than of the other methods, and the FDR of our BHM was lowest in most of the scenarios. When under-dispersion is present in the data **(B)** the FDR of EdgeR is still high, the FDR of the BHM does increase with a low number of subjects. (PR: phylogenetic relationships)

THE PERFORMANCE MEASUREMENTS FOR THE BAYESIAN MODELS, THE STANDARD NB REGRESSION MODEL, THE STANDARD ZERO-INFLATED NB REGRESSION MODEL AND THE POISON MODEL

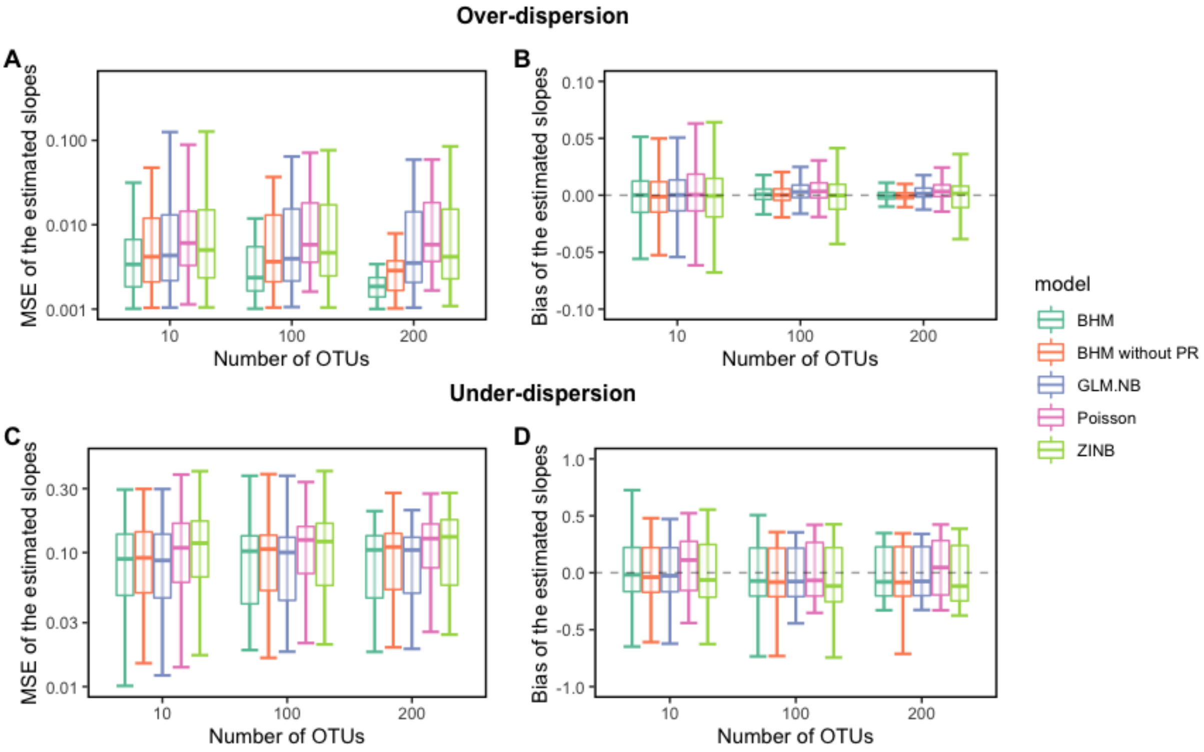

Figure S2. The comparison between the true slope and the estimated slope of the simulation study; the mean squared error (MSE) (**A**,**C**) and bias between the slopes (**B**,**D**). (PR: phylogenetic relationships)

FULL COMPARISON BETWEEN METHODS OF DIET-MICROBE ASSOCIATIONS IN THE HELIUS DATA

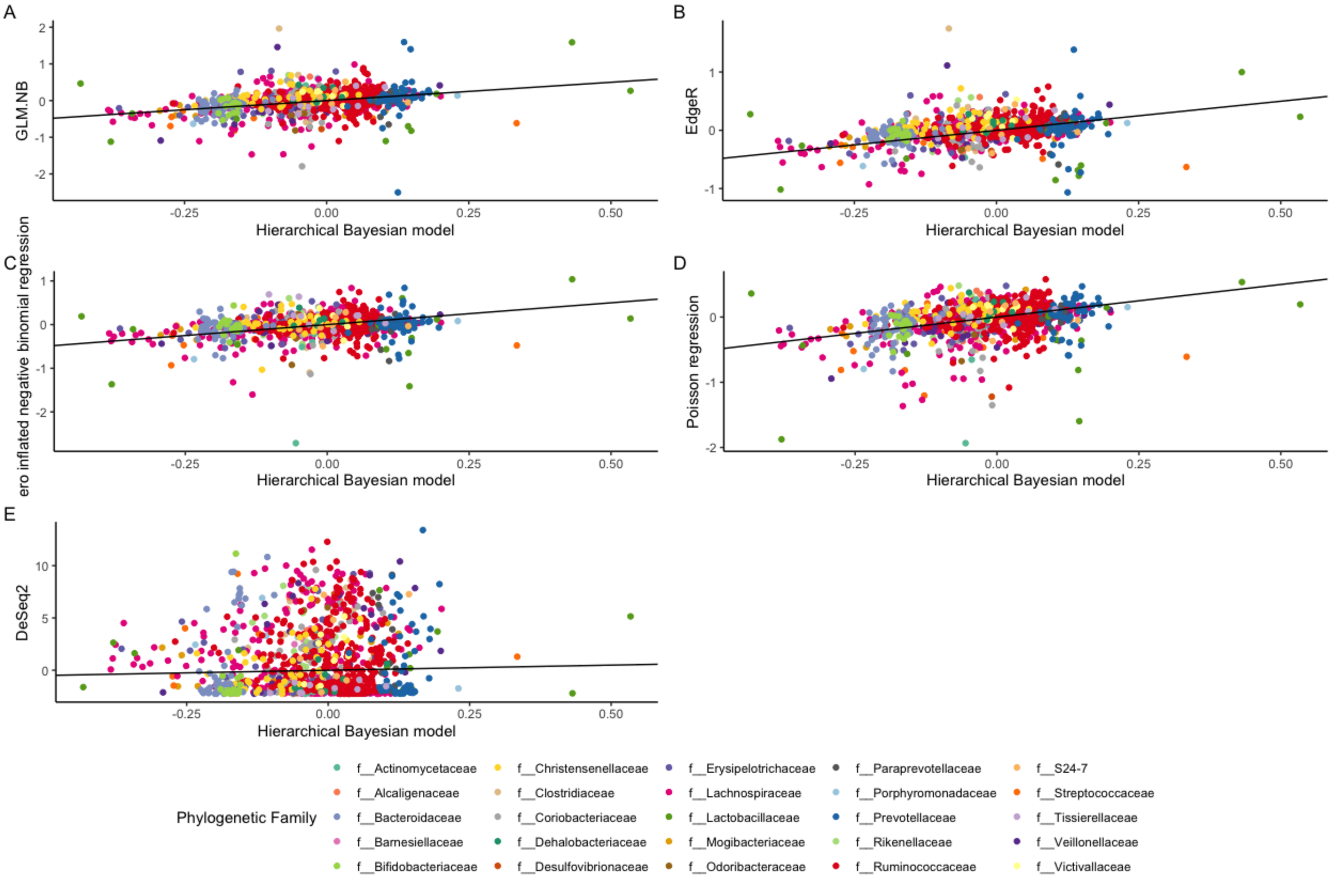

Figure S3. Comparison of the estimated associations between the **(A)** standard NB regression model (GLM.NB), **(B)** EdgeR, **(C)** zero-inflated NB regression model, **(D)** Poisson regression, **(E)** DeSeq2 and the Bayesian Hierarchical model (BHM) with phylogenetic based shrinkage for the HELIUS study data.

CORRELATION CLUSTERING

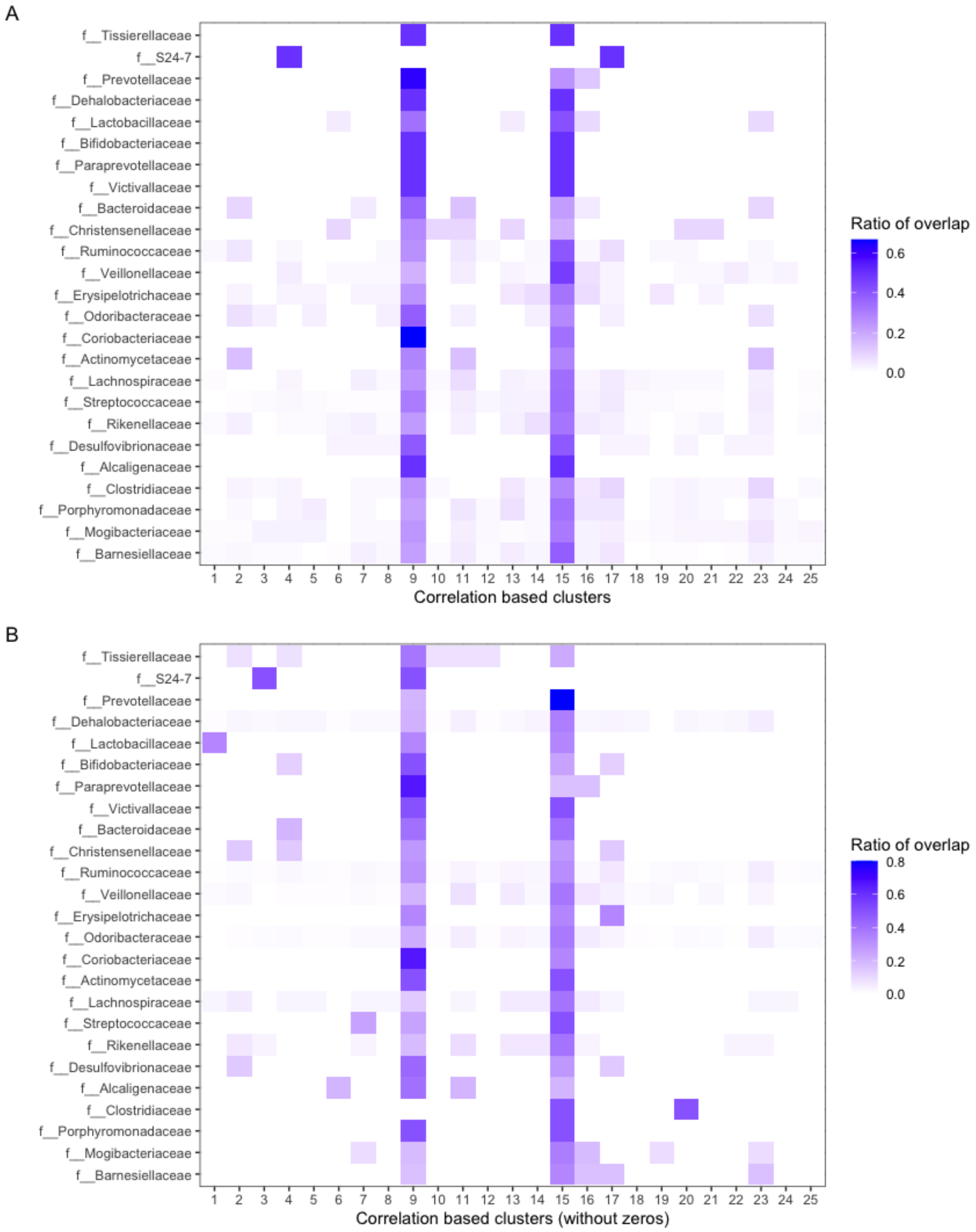

Figure S4. The overlap between the phylogenetic families and correlation-based clustering of the count data of the HELIUS study. The overlap ratio is defined as the number of OTUs per family in a cluster divided by the total number of OTUs in that cluster. In **A** the correlations are calculated with the inclusion of all zeros in the count data. In **B** the correlations are calculated based on the method proposed by Bichat *et al.*(2020), excluding shared zero counts between the OTUs.

USING GENUS LAYER AS SECOND LAYER IN MODEL

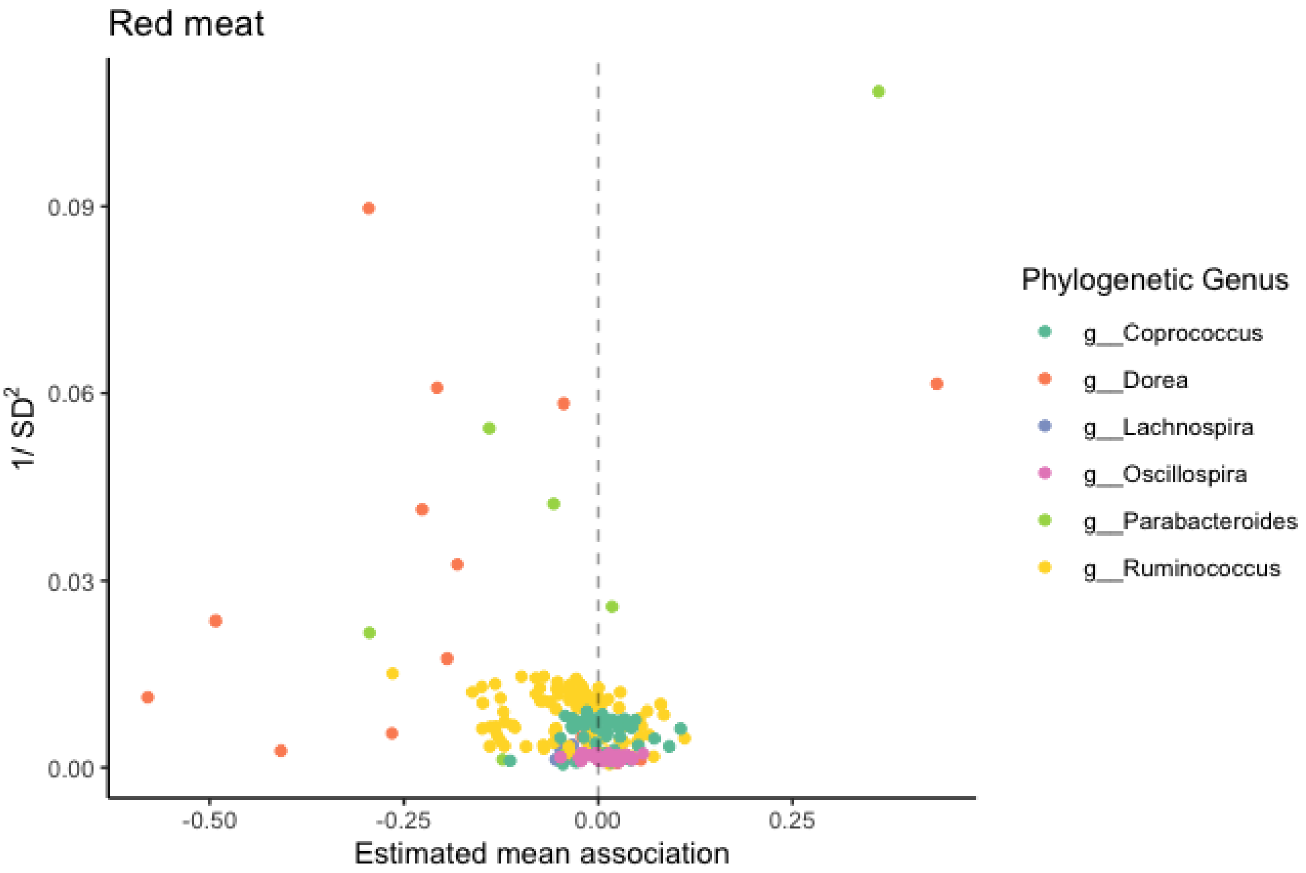

Figure S5. Volcano plot of the diet-microbe associations between the FFQ scores of red meat, using the Genus layer of the phylogenetic relationships as classification.
